# Hedgehog signalling in liver regeneration

**DOI:** 10.1186/2047-783X-19-S1-S4

**Published:** 2014-06-19

**Authors:** Anna Diehl

**Affiliations:** 1 Department of Medicine, Duke University, Durham, North Carolina, 22210, USA

## Introduction

The adult liver has unique regenerative capabilities. Adult livers generally regenerate fully functional liver cells when injured, unlike other vital adult organs which typically respond to cell loss by forming scar tissue. The mechanisms underlying these differences are not well understood, particularly since adult livers are perfectly capable of scarring. In fact, transient scar formation normally occurs in situations that lead to efficient and complete recovery of functional liver mass, such as 70% partial hepatectomy (PH). Scarring also occurs to some extent during many types of chronic liver injury, although progressive scarring (dubbed cirrhosis) occurs in only a minority of individuals with chronic liver injury. Even in cirrhotic livers, however, scarring has been shown to regress gradually once factors driving chronic injury are alleviated. The aggregate data suggested to us that the unique regenerative capabilities of adult livers are linked to its ability to control the formation and regression of scar. Our research has focused on delineating the mechanisms that control scarring. The results will help to clarify why injured livers typically regenerate healthy parenchyma, as well as why other organs replace wounded tissue with scar.

## Hedgehog controls liver scarring and regeneration

The wound healing process necessitates some degree of scarring. Scarring is characterized by excessive extracellular matrix deposition, but also involves inflammation, vascular remodeling, and accumulation of cell types that are relatively inconspicuous in healthy tissues, such as progenitors and myofibroblasts (MF). In adult livers, each of these scarring-related responses is regulated by Hedgehog (Hh), a fetal morphogenic signaling pathway that reactivates during adult liver injury [1]. Hh pathway activity is barely detected in healthy adult livers, increases during chronic injury in parallel with the intensity of scarring, and subsides when injury dissipates and scarring regresses. Regression of scarring can be induced in mice with advanced liver fibrosis by treating them with a Hh inhibitor, proving that adult livers require active Hh signaling to sustain scarring. Also, just as transient scarring is necessary for the liver to regenerate after acute injury, transient Hh pathway activation is required for normal liver regeneration after PH. Treating healthy mice with a drug that blocks Hh signaling not only inhibits scar formation but also reduces proliferation of liver epithelial cells, liver mass recovery, and survival after PH [2]. Although these data identify Hh as a critical regulator of liver scarring and thereby, regeneration, the mechanisms involved remain uncertain.

## Hedgehog regulates scarring and regeneration of chronically injured livers by controlling the fate of multipotent progenitors

Recently, we proved that canonical Hh signaling controls the fate of liver MF [3]. MF accumulation is a hallmark of scarring in all tissues. In chronically injured adult livers, many of the MF that accumulate are derived from resident hepatic stellate cells (HSC). We showed HSC produce Hh ligands (Sonic Hh, Shh), express the Hh co-receptors, Patched (Ptc) and Smoothened (Smo), activate Hh-regulated Gli-family transcription factors, and induce expression of various Gli-target genes as they transition to become MF during culture and during liver injury. Moreover, we proved that conditionally deleting Smo in HSC-derived MF during culture and during liver injury abrogated Hh signaling, and caused the cells to down-regulate their expression of multiple MF genes and re-acquire a phenotype that was more typical of quiescent HSC. This led to scar regression, but also reduced hepatocyte and cholangiocyte proliferation and caused relative liver atrophy, confirming that appropriate control of Hh signaling is essential for normal liver regeneration [3]. Subsequent lineage tracing studies demonstrated that cells expressing HSC markers generated hepatocytes and cholangiocytes during chronic liver injury. Although both quiescent and MF-HSC expressed Sox9, a marker of bipotent liver epithelial progenitors, HSC expressing the MF marker, alpha smooth muscle actin (aSMA) were particularly enriched with markers of multi-potent progenitors, such as Nanog, Oct4, Cd44, and Cd24 [3]. Based on these findings, we forwarded the hypothesis that adult HSC-derived MF are Hh-regulated, multi-potent progenitors.

## Hedgehog-regulated multi-potent progenitors regenerate adult livers after PH

Recently, we have begun to evaluate a related novel hypothesis, namely, that Hedgehog controls liver regeneration after PH by modulating the differentiation of these multi-potent myofibroblastic progenitor cells. Transgenic mice were generated to permit conditional deletion of either floxed-Smoothened or floxed-YFP alleles in aSMA-expressing cells. By studying the Smoothened-deleted mice, we were able to determine how selectively abrogating Hh signaling in aSMA(+) cells (and their progeny) influenced regenerative responses to PH. By studying the YFP-marked mice, we were able to track the fate of cells that expressed aSMA after PH. The results demonstrate that Hh signaling in aSMA(+) cells orchestrates both scarring and regeneration after PH; prove that these processes are required for the liver to regenerate normally after PH; and show that post-PH regeneration involves differentiation of myofibroblastic progenitors into hepatocytic and ductular cells [4].

## Summary

Regeneration of adult livers requires Hh-dependent modulation of epithelial-mesenchymal transitions in multipotent progenitors. Data generated by studying models of acute and chronic liver injury reveal that robust epithelial to mesenchymal transitions (EMT) normally occur in injured livers, and show that conditionally abrogating canonical Hedgehog signaling in multi-potent aSMA-expressing cells blocks these EMT, instead triggering a cascade of mesenchymal-to-epithelial transitions (MET). This has global consequences for liver wound healing. Regeneration of different types of liver cells becomes unbalanced, leading to excessive accumulation of quiescent HSC but reduced outgrowth of hepatocytic and ductular progenitors and their progeny, as well as depletion of MF populations. Thus, de-regulating Hh-sensitive EMT/MET responses disrupts both scarring and regeneration of injured livers. These findings, in turn, suggest that the liver is uniquely suited for regeneration because it harbors large populations of multi-potent progenitors and is generally able to constrain signals, such as Hedgehog, that modulate the differentiation of these cells towards less epithelial (and more mesenchymal) phenotypes.
